# Monoclonal Antibodies as Diagnostics; an Appraisal

**DOI:** 10.4103/0250-474X.62229

**Published:** 2010

**Authors:** M. Z. Siddiqui

**Affiliations:** Processing and Product Development Division, Indian Institute of Natural Resins & Gums, Namkum, Ranch-834 010, India

**Keywords:** Antibody engineering, applications, diagnostics, enzyme-linked immunosorbent assay, immunoassay, monoclonal antibodies, therapeutics

## Abstract

Ever since the development of Hybridoma Technology in 1975 by Kohler and Milstein, our vision for antibodies as tools for research for prevention, detection and treatment of diseases, vaccine production, antigenic characterization of pathogens and in the study of genetic regulation of immune responses and disease susceptibility has been revolutionized. The monoclonal antibodies being directed against single epitopes are homogeneous, highly specific and can be produced in unlimited quantities. In animal disease diagnosis, they are very useful for identification and antigenic characterization of pathogens. Monoclonal antibodies have tremendous applications in the field of diagnostics, therapeutics and targeted drug delivery systems, not only for infectious diseases caused by bacteria, viruses and protozoa but also for cancer, metabolic and hormonal disorders. They are also used in the diagnosis of lymphoid and myeloid malignancies, tissue typing, enzyme linked immunosorbent assay, radio immunoassay, serotyping of microorganisms, immunological intervention with passive antibody, antiidiotype inhibition, or magic bullet therapy with cytotoxic agents coupled with anti mouse specific antibody. Recombinant deoxyribonucleic acid technology through genetic engineering has successfully led to the possibility of reconstruction of monoclonal antibodies viz. chimeric antibodies, humanized antibodies and complementarily determining region grafted antibodies and their enormous therapeutic use.

Antibodies are a mixture of closely related immune system proteins, with subtle but important differences. Conventional techniques for preparation of antibodies in antiserum against a particular antigen always result in the production of antibodies having different classes, specificities and affinities. The development of Hybridoma Technology by Kohler and Milstein[1,2] has provided the means to partition the complex antibody responses into their individual components. That's why monoclonal antibodies (MAbs) have revolutionized the diagnostic science with their specificity towards specific antigen and almost unlimited production. MAbs can be produced to virtually any antigen, they are completely homogeneous populations and entail fewer problems of cross reactivity than are often observed with conventional polyclonal antibodies[2].

The development of hybridoma technology offered a number of advantages over the original art of polyclonal antibodies viz. (i) the generation of MAbs is now a standard and increasingly routine procedure, (ii) the use of impure antigen is tolerated since the detection of MAb of interest is largely dictated by the selection strategy, (iii) antibodies with selected properties or reactivities for specific structures could be selected and (iv) since the hybridoma cells line is immortal, there is an unlimited source of the MAb[2].

In hybridoma technology, a myeloma cell rendered drug sensitive through mutation in a growth essential gene hypoxanthine guanine phosphoribosyl transferase (HGPRT) is chemically fused with immune cells from a host immunized with the antigen of interest and the resulting cells are grown in medium containing the selective drug. Since the immune cells have a short life span in tissue culture and the myeloma cells are drug sensitive, the only cell that will survive are those myeloma cells which obtained a normal HGPRT gene from the immune cells. Such cells also have a high likelihood of carrying the immune cell's antibody gene resulting in the generation of a hybridoma that can grow continuously *in vitro* and secrete a single monoclonal antibody[1].

Selection of hybridomas secreting desired antibodies is quite tedious. Screening assay should be rapid, reliable, versatile, sensitive and easily performed with a large number of samples. The most commonly used system fulfilling these criteria is the enzyme linked immunosorbent assay (ELISA). However, antibodies can also be detected by radio immunoassay (RIA), immune fluorescence and haemolytic plaque assays. In ELISA, an antigen is passively adsorbed to a plastic surface (polystyrene). The sample (serum/culture supernatant) is applied to the immobilized antigen. The monoclonal antispecies antibody enzyme conjugate is then added, followed by addition of substrate and spectroscopy. Selected hybridomas after screening are expanded to be frozen and cloned to generate monoclonal by limiting dilution method. The selected and desired MAbs can be purified by affinity chromatography, being more efficient and less tiresome. They are characterized by isotyping to determine the class/sub class of immunoglobulins by ELISA and western blot to test their ability to bind different antigen preparations.

Since the inception of hybridoma technology, various approaches and technologies have been developed for large scale production of MAbs. For a long time, the *in vivo* production in mice by ascites induction has been preferred for its cost effectiveness and high concentration of MAbs produced. But the growing ethical concern about mice and the improved cell culture techniques, led to an increased emphasis on *in vitro* methods being parallel to the *in vivo* methods both in capacity and cost effectiveness[3]. Conventional low cell density culture methods result *in vitro* production of MAbs which are released in culture medium at concentrations between 1 to 100 μg/ml[4]. In the recent past, efforts have been made to design high density culture systems, leading to the development of various bioreactors. They can generate high yields of MAbs (100 mg/week on an average), but only allow the production of one MAb at a time and suffer the disadvantage of expense, complexity and proneness to contamination[5]. But Trebak *et al*.[3] have demonstrated that the cell line culture flasks permit long term culture of hybridoma cells in high density allowing abundant production of highly active and pure MAbs necessary for specific purposes.

During the past decades MAbs have found tremendous applications in therapeutics, drug targeting and had a profound impact on diagnostics. The diagnosis of any infectious disease often needs the demonstration of the causative organism or a specific antibody. In some infectious diseases such as reproductive or respiratory infections, the causative organism can be demonstrated throughout the course of infection. Specific antibody based tests identify the pathogens associated with the disease. MAbs recognizing unique antigenic determinants on pathogens can be developed. MAb can read with a single antigenic determinant (epitope) and this restricted reactivity allows for precise identification of the organism of interest which is the major advantage of MAbs over polyclonal antisera. In case of a pathogen occurring as sub type defined by unique antigenic differences, specific MAbs can be used whereas conventional antisera needs laborious absorption to remove cross reactive antibodies. Because of the specificity, homogeneity and unlimited availability of the MAbs, vast amount of work has been carried out on the production/development of MAbs diagnostic reagent tests against various pathogenic agents of veterinary importance.

Production of monoclonal antibodies against mouse lymphocyte surface glycoprotein has been reported by Trowbridge[6], against human lymphocyte by Trucco *et al*.[7], against major histocompatibility antigens by Galfre *et al*.[8] and for analysis of human leucocyte antigen (HLA) system by Brodsky *et al.*[9]. Falmand *et al*.[10] have used monoclonal antibodies in the detection of antigenic differences between rabies and rabies related virus proteins I, the nucleocapsid protein, and Yewdell and Gerhard[11] for antigenic characterization of viruses. Shigeo *et al*. in 1982[12] and Weis *et al*. in 1983[13] have reported the production and characterization of monoclonal antibodies against 10 S DNA polymerase α from calf thymus and avian retrovirus reverse transcriptase, respectively.

The immuno diagnosis of protozoal and parasitic diseases has significantly been improved by MAb technology because the tests involving MAb as diagnostic reagents overcome the limitations of polyclonal antibodies. MAbs were found extremely useful in the rapid outbreak of East Coast Fever (ECF)[14]. MAbs of diagnostic value have also been developed against *Trichomonas vaginalis*[15], *Leishmania donovani*[16], *Trypanosoma congolense*[17], *Babesia bovis*[18]. With improved sensitivity and specificity of the diagnostic test system, MAbs have also been developed against a number of animal viruses viz., bovine herpes virus, cervine herpes virus type I, pseudo rabies virus, calf strain RIT 4237 (sub-group I) and human strain 82-561 (sub group 3) of rotavirus[19–21]. The possibilities of using MAbs to CEA in scintigraphic diagnosis of tumors have been reported by Steenbeck and Markwardt in 1985[22] and their use in diagnostics and therapy of allergic diseases by Becker and Schlaak in 1989[23]. Production and characterization of MAbs as diagnostics for fibrinogen fibrin and *P. carinii* pneumonia have been reported by Kudryk *et al.*[24] and Nato *et al.*[25], respectively. Development of monoclonal antibodies for the detection of Mycoplasma pneumonia and plum pox virus has been reported by Geary *et al*[26] and Hilgert *et al*[27], respectively.

Taylor *et al.*[28] have compared high performance liquid chromatography (HPLC) and monoclonal fluorescence polarization immunoassay (mFPIA) for the determination of whole blood cyclosporin A in liver and heart transplant patients and they have found that mFPIA is not interchangeable with HPLC. A rapid and simple ‘naked eye’ dipstick system was developed to detect human rotavirus in faeces, using nitrocellulose as solid phase, two MAbs, and colloidal gold as marker. For human RV the specificity and sensitivity was 100% when compared with a commercial latex system, and 99% and 98%, respectively, when correlated with traditional RNA-PAGE, and 100% and 98% when compared to an ELISA system[29].

Use of MAbs with magnetic particles to separate cell sub populations by negative selection as well as by positive selection has been reported by Vaccaro and Markenacin 1995[30,31]. Quiliz *et al*.[32] have compared an acid fast stain and a monoclonal antibody based immunofluorescence reagent for the detection of Cryptosporidium oocysts in faecal specimens from cattle and pigs. Laurino *et al*[33] have stated that the development of MAbs based immunochemical assays to measure antibodies, antigens and small molecules, such as drugs, ushered in a revolution in modern diagnostic medicine and their impact on diagnostic imaging technologies and therapeutic regimes was equally dramatic. Generation and characterization of MAb against cell wall extracts of *Candida albicans* ATCC 26555 as an immunoglobulin G1 used for the recognition of high molecular mass proteins present in the cell wall of *Candida albicans* has been reported by Marcilla *et al*[34].

Two easy-to-use commercial diagnostic assays, a dipstick ELISA and an immuno chromatographic card assay were evaluated for detection of IgM antibody to dengue virus with an in house IgM antibody capture microplate ELISA as a reference assay. Both commercial assays provide sensitive and specific detection of anti dengue virus IgM antibody and would prove useful in settings where the microplate ELISA is impractical[35].

Monoclonal and recombinant antibodies provide a never ending source of molecules and can produce endless possibilities for novel genetic constructs. Antibodies are still very much in vogue and are now also being used in micro array analysis of the proteome using protein chips[36].

Skin derived anti leukoproteinase (SKALP), also known as elafin, is an epithelial proteinase inhibitor with antimicrobial properties that is not normally expressed in human epidermis, but is induced under inflammatory conditions and in some types of skin cancer[37]. SKALP is a member of the recently described trappin gene family, which encodes a new class of proteins, characterized by a transglutaminase substrate domain. Vandermeeren *et al*.[37] have developed ten different mouse MAbs recognizing an epitope in the proteinase inhibiting domain. The antibodies could be used with high specificity by immuno histochemistry on formalin fixed tissue, by affinity chromatography, by Western blotting and by ELISA for detection of SKALP/elafin. These antibodies have several advantages over existing polyclonal antisera, such as defined epitope, the detection of full length SKALP/elafin and unlimited supply. An antibody against the hexapeptide epitope, which is common to all known simian, bovine, and swine trappin family members, was used to immuno localize bovine trappins expressed in tracea, that have recently been discovered. These MAbs will serve as important new tools to measure SKALP/elafin and trappin family members in research and diagnostics. Similarly a MAb (R5) that recognizes a potential celiac toxic repetitive pentapeptide epitope in gliadins has been reported by Osman *et al*[38].

Monoclonal antibodies based competitive immunoassay for the detection of specific antibodies to *Y. pseudotuberculosis* has been developed by Fedorova *et al*[39]. Peknicova *et al*.[40] have developed MAbs against sperm intra acrosomal antigens as markers for male infertility diagnosis and estimation of spermatogenesis. Indigenously produced potent MAbs against A, B and H blood group antigens used as diagnostics have been reported by Iyer *et al*[41].

The diversity of antibody engineering technologies is amazing; synthetic combinatorial libraries, cell free libraries, *in vitro* affinity maturation, large scale production of antibodies in transgenic animals and plants, chimeric and humanized antibodies, human MAbs from transgenic mice using conventional hybridoma techniques, intrabodies and so many. These and similar constructs will provide the basis of an incredible number of new therapeutic antibody based products, besides being a transition to less costly and smaller synthetic molecules like bioactive peptides etc. The time and cost of travelling from the laboratory to the clinic are shorter for MAbs than for many conventional drugs. Antibodies are relatively easy to detect, manipulate and test. The FDA now regards MAbs as biotechnology derived pharmaceuticals with relatively few reports of serious side effects. The public health possibilities of MAbs are fabulous. Additional MAbs are being investigated in treatment of a variety of cancers including B-cell lymphomas, multiple myeloma and colorectal cancer besides rheumatoid arthritis, allergic asthama and others characterized by chronic inflammation[42,43]. Since the route of entry of many diseases is through the mucosal membranes, therefore, a MAb delivered to a mucosal surface can provide immediate protection against infection. The monoclonal antibodies that are licensed to treat other diseases, such as cancer autoimmune diseases, are being tested for the treatment of multiple sclerosis (MS). In fact, MAbs are now among the most promising therapies for MS[44]. The pharmacokinetics and pharmacodynamics of monoclonal antibodies have been reported by Wang *et al*[45]. Using a modified two step screening procedure, production of ultrasensitive generic monoclonal antibodies against major aflatoxins have been reported by Daohong *et al*[46]. Very recently a refined surface plasmon resonance technique has been adopted for the screening and selection of monoclonal antibodies by Matthew *et al*[47].

The basic mechanism of a MAb is the same as an antibody produced by the body. However, when MAbs are used in the diagnosis and treatment of diseases, certain substances are often added to them to give them their therapeutic and diagnostic characteristics. They can also be used on their own to block or encourage certain responses from the immune system. In diagnosis, radioactive markers are attached to them to locate a certain kind of cell within the body. They are used in diagnostic imaging of internal organs and tumors.

While the utility of MAbs as drugs that can specifically target a disease associated antigen is enormous, it has taken a quarter century for these molecules to be adopted as bonafide diagnostics. Despite their slow pace in drug development during the pioneering years, it is now estimated that there are nearly 500 MAb based therapies in the field. Major factors that have influenced the acceptance of MAbs as diagnostics include their drug safety profiles, technological advancements for facilitating MAb's discovery, their development and market success.

Monoclonal antibodies generally exhibit exquisite specificity for the target antigen. The binding site on the antigen called the epitope, can be linear or conformational, and may comprise continuous or discontinuous amino acid sequences. The epitope is the primary determinant of the antibody's modulatory functions and depending on the epitope, the antibody may exert antagonist or agonist effect, or it may be non modulatory. The epitope may also influence the antibody's ability to induce antibody dependent cell mediated cytotoxicity (ADCC) and complement dependent cytotoxicity (CDC). MAbs exert their pharmacological effects via multiple mechanisms that include direct modulation of the target antigen, CDC, ADCC, and delivery of a radio nucleotide or immuno toxins to target cells[48].

## CONCLUSION

The monoclonal antibody production technology has revolutionized the world of Biotechnology. Advances in genetic engineering over the years have provided numerous ways to design MAbs that are more robust and efficacious compared with their original murine version. MAbs have not only been used as diagnostics, therapeutics, research reagents, drug targettor for various infectious diseases but also cancerous, metabolic and hormonal disorders. MAb technology in conjunction with recombinant DNA technology has successfully led to the reconstruction of chimeric antibodies, humanized antibodies and CDR grafted antibodies and has enormous potentials for therapeutic uses.
